# Luteolin-loaded exosomes derived from bone marrow mesenchymal stem cells: a promising therapy for liver fibrosis

**DOI:** 10.1080/10717544.2022.2142700

**Published:** 2022-11-03

**Authors:** Asmaa A. Ashour, Amal H. El-Kamel, Radwa A. Mehanna, Ghada Mourad, Lamia A. Heikal

**Affiliations:** a Department of Pharmaceutics, Faculty of Pharmacy, Alexandria University, Alexandria, Egypt; b Department of Medical Physiology, Faculty of Medicine, Alexandria University, Alexandria, Egypt; c Center of Excellence for Research in Regenerative Medicine and Applications (CERRMA), Faculty of Medicine, Alexandria University, Alexandria, Egypt; d Department of Histology and Cell Biology, Faculty of Medicine, Alexandria University, Alexandria, Egypt

**Keywords:** Phytomedicine, exosomes–antifibrotic activity, mesenchymal stem cells, inflammatory cytokine, profibrotic markers

## Abstract

Liver fibrosis is a global life-threatening disorder with no approved treatment. It leads to serious hepatic complications when progressive, such as cirrhosis and carcinoma. Luteolin (LUT) is a plant flavonoid possessing a promising therapeutic potential in various liver diseases particularly, liver fibrosis. It was reported to have potent anti-inflammatory and antioxidant properties. It also suppresses the proliferation of activated hepatic stellate cells (HSC) and induces their apoptosis. However, its poor aqueous solubility and exposure to metabolism hinder its clinical use. Mesenchymal stem cells (MSCs)-derived exosomes, nano-sized extracellular vesicles, have recently emerged as natural biocompatible drug delivery vehicles permitting efficient drug delivery. Accordingly, the present study aimed for the first time to investigate the potential of bone marrow MSCs-derived exosomes to improve LUTs antifibrotic effectiveness. LUT-loaded exosomes (LUT-Ex) were successfully developed, optimized and subjected to both *in vitro* and *in vivo* characterization. The elaborated LUT-Ex presented good colloidal properties (size; 150 nm, PDI; 0.3 and ζ-potential; −28 mV), typical vesicular shape, reasonable drug entrapment efficiency (40%) with sustained drug release over 72 h. Additionally, the cellular uptake study of coumarin-6-loaded exosomes in HEP-G2 revealed a significant enhancement in their uptake by 78.4% versus free coumarin-6 solution (*p* ≤ 0.001). Following a single intraperitoneal injection, LUT-Ex revealed a superior antifibrotic activity compared with either LUT-suspension or blank exosomes as evidenced by the results of biochemical and histopathological evaluation. In conclusion, the elaborated LUT-Ex could be addressed as a promising nanocarrier for effective treatment of liver fibrosis.

## 1. Introduction

Liver fibrosis is a frequent and potentially life-threatening disorder with global domination. The consequence of progressive fibrosis is cirrhosis, which often leads to the development of hepatocellular carcinoma (HCC) and end-stage liver failure. Fibrogenesis is considered as an abnormal wound-healing response to liver injury. Following hepatocellular injuries, many fibrogenic pathways are activated which stimulate the formation of an extracellular matrix, made of different collagen types, by the activated hepatic stellate cells (HSC) (Acharya et al., 2021).

Up till now, there is no treatment for liver fibrosis and elimination of the etiological factors represents the adopted strategy for therapy. Currently, many antifibrotic drugs are in different phases of clinical trials and none of them was finally approved for clinical use (Ashour et al., 2021). This necessitates finding new therapeutic options for effective disease management.

In line with that, many phytomedicines have long been exploited to treat liver fibrosis as an alternative natural approach and among them Luteolin (LUT) exerts a surpassing activity (Domitrović et al., 2009; Li et al., 2015). Luteolin, 3′,4′,5,7-tetrahydroxyflavone, is a flavonoid found in many edible plants. Besides being a potent anti-inflammatory (Balamurugan & Karthikeyan, 2012; Tai et al., 2015; Park & Song, 2019), antioxidant agent (Balamurugan & Karthikeyan, 2012), it can alleviate liver fibrosis via suppressing proliferation of activated HSC (Li et al., 2015; Cummins et al., 2018). Additionally, it can induce apoptosis for the stimulated HSC (Li et al., 2015). In spite of its therapeutic benefits, its clinical utility is hindered by its low bioavailability being of poor aqueous solubility as well as its exposure to extensive metabolism (Dang et al., 2014; Liu et al., 2014; Elnaggar et al., 2018; Luo et al., 2019).

Numerous research studies were conducted to enhance LUT bioavailability based on the use of various nanocarriers (Dang et al., 2014; Liu et al., 2014; Elnaggar et al., 2018; Luo et al., 2019), but none of them addressed the use of exosomes as naturally-equipped nanosystems having interesting pharmacological activities.

Exosomes, nanosized extracellular vesicles, have recently emerged as bioinspired drug delivery vehicles permitting for targeted drug delivery (Batrakova & Kim, 2015; Patil et al., 2020). Using exosomes in drug delivery has captured the interest of researchers as they combine the benefits of both cell-based delivery systems and nanotechnology (Batrakova & Kim, 2015). Exosomes are advantageous over other synthetic nanocarriers owing to circumventing the issues of rapid clearance and toxicity (Batrakova & Kim, 2015). As well, they are preferred over their parental cells due to easier handling, higher stability, lower chance for immunological rejection and no risk for tumorigenesis (Thabet et al., 2020).

Exosomes isolated from various mesenchymal stem cells (MSCs) have gained popularity for treatment of many hepatic disorders as they have the same regenerative power of their parental cells. In light of this, the efficacy of adipose MSCs-derived exosomes to deliver microRNA to HSC was proven to alleviate carbon tetrachloride (CCl_4_)-induced liver fibrosis (Qu et al., 2017). Moreover, exosomes isolated from amnion-MSCs were reported to alleviate liver inflammation and fibrosis in rats (Ohara et al., 2018). Similarly, the hepatoprotective effect of bone marrow and umbilical cord MSCs-derived exosomes was investigated using different animal models (Li et al., 2013; Jiang et al., 2018; Shao et al., 2020). They were reported to have an antioxidant activity (Jiang et al., 2018) as well as having the ability to decrease liver inflammation via suppression of macrophages activation (Shao et al., 2020).

Based on the aforementioned background, the present study aimed at exploring the ability of bone marrow MSCs derived-exosomes to augment the antifibrotic efficacy of LUT. LUT-loaded exosomes were prepared and *in vitro* characterized for the first time. Furthermore, their *in vivo* efficacy in resolution of the CCl_4_-induced liver fibrosis was evaluated both biochemically and histopathologically.

## 2. Materials and methods

### 2.1. Materials

Luteolin (purity 98%) was purchased from Baoji Guokang Bio-Technology Co., Ltd China. Absolute ethanol, carbon tetrachloride 98% and liquid paraffin were purchased from ADWIC, El-Nasr Pharmaceutical Chemicals Co, Egypt. Coumarin-6, Dulbecco’s modified eagle medium (DMEM) and fetal bovine serum (FBS) were obtained from Sigma-Aldrich, USA. Alanine aminotransferase (ALT), aspartate aminotransferase (AST) assay kits were purchased from Spectrum, Germany. Alkaline phosphatase (ALP) activity kits were purchased from Abcam, USA. Rat tumor necrosis factor-α (TNF-α) ELISA kits were purchased from R&D Systems Inc., Germany. Rat matrix metaloproteinase-2 (MMP-2) and transforming growth factor-β (TFG-β) enzyme-linked immunosorbent assay (ELISA) kits were purchased from Novus biologicals, USA. Hydroxyproline (HP) colorimetric assay kit was purchased from Biovision, USA. All other reagents and chemicals were of analytical grade.

### 2.2. Methods

#### 2.2.1. Bone marrow MSCs isolation, culture and characterization

The cells were obtained from male Sprague–Dawely rats as described previously by Abolgheit et al. (2021).

Rats’ femurs and tibias were dissected aseptically, their ends were trimmed, and the bone marrow was flushed with complete media by inserting a 23-gauge needle attached to a 5 mL syringe into a 50 mL falcon tube. Flushed marrow was then re-suspended and passed through a 70-mm filter mesh in a new falcon tube to remove any bone spicules or cell clumps. The cell suspension was centrifuged at 1,200 rpm for 5 min then resuspended in complete media, DMEM Minimum Essential Medium L.G supplemented with 10% fetal bovine serum (FBS), 1% penicillin/streptomycin (P/S, 10,000 IU/mL/10,000 μg/mL, Lonza) plated in T-25 flask and cultured in humidified 5% CO_2_ incubator at 37 °C. The medium was changed after 48 h, the nonadherent cells were removed, then changed every 2–3 days and follow-up of cultured cells was done using a phase-contrast inverted microscope equipped with a digital camera (Olympus CKX41SF, Japan).

When adherent cells reached 80% confluence, they were washed with phosphate buffer saline (PBS) then treated with 0.25% trypsin/ethylene diamine tetra-acetic acid (EDTA) solution to dissociate then split the cells in a ratio of 1:3.

Immunophenotyping was done to the cells at passage three (P3) for the expression of mesenchymal and hematopoietic cell surface markers using fluorescent-labeled monoclonal antibodies (mAb) for CD44, CD90, CD73, CD105, CD11b and CD45. Adherent cells were trypsinized, washed with PBS and incubated, at room temperature for 30 min in the dark, with monoclonal allophycocyanin-conjugated anti-CD90, at a concentration of 30 µg/mL (anti-Thy1.1) (Abcam, Cambridge, UK), monoclonal phycoerythrin (PE) -conjugated anti-CD45, at a concentration of 200 µg/mL (Abcam, Cambridge, UK), monoclonal PE-conjugated anti-CD73, at a concentration of 100 µg/mL (Abcam, Cambridge, UK), monoclonal unconjugated antibody for CD105, at a concentration of 1 mg/mL (Abcam, Cambridge, UK), monoclonal PE-conjugated antibody for CD11b (0.2 μg/mL, Abcam, Cambridge, UK) and anti-CD44 PE-conjugated antibody at a concentration of 100 µg/mL (Abcam, Cambridge, UK).

Immunofluorescence on the viable cells was performed using BD FACS Calibur flow cytometer equipped with Cell Quest software (Becton Dickinson, New Jersey, USA). Characterized P3 cells were then used for the isolation of exosomes.

#### 2.2.2. Isolation of exosomes (Ex)

At passage 3, bone marrow-MSCs were grown in serum-free conditioned medium for 48 h. The conditioned media was centrifuged at 300×*g* for 5 min to remove any cellular debris. The obtained supernatant was further centrifuged, at 16,500×*g* for 40 min at 4 °C, then filtered using 0.2 µm Millipore filter aiming to eliminate any large microvesicles. Finally, the exosomes were separated by subjecting the supernatant to ultracentrifugation at 120,000×*g* for 70 min at 4 °C followed by their redispersion in phosphate buffer saline (PBS) (Abolgheit et al., 2021).

#### 2.2.3. Preparation of luteolin-loaded exosomes (LUT-Ex)

For luteolin loading into exosomes, the method previously reported by Kim et al. was used with slight modifications (Kim et al., 2016). Briefly, equal volumes of 100 µL from both exosomes and luteolin solution (7 mg/mL in methanol) were mixed in 1 mL PBS. For optimization purpose, two methods of drug loading were tried; the sonication and the physical incubation method. Concerning the sonication method, drug loading into exosomes was achieved by subjecting drug–exosomes mixture to ultrasonication using a probe sonicator (Bandelin sonoplus TS103, Germany) with the following settings: 20% amplitude, 10 cycles of 3 s on/off for 3 min with 2 min cooling period in an ice bath between each cycle. After sonication, the prepared LUT-Ex were incubated at 37 °C for 1 h to allow for recovery of the exosomal membrane. Whereas in the physical incubation method the LUT solution was incubated with exosomal dispersion at 37 °C for 1 h with shaking (Kim et al., 2016).

#### 2.2.4. *In vitro* characterization of blank and LUT-Ex

Isolated exosomes were analyzed according to their surface tetraspanin proteins using a modified protocol (Barranco et al., 2019). Conjugated antibodies against CD9, CD63, and CD81 were used to label the exosomes. 50 μL of Exosome MicroBeads was added to the exosome sample, vortexed and incubated for 1 h at room temperature. Following incubation exosome bound beads were further washed in PBS/1% BSA, blocked with 10% BSA, and stained with Anti-CD9 [CD9 (Santa Cruz Biotechnology, (C-4):sc-13118) conjugated with AlexaFlour@488, Anti-CD63 Antibody (Santa Cruz Biotechnology, MX-49.129.5: sc-5275) conjugated with AlexaFlour@647, and Anti-CD81 Antibody (Santa Cruz Biotechnology, (1.3.3.22): sc-7637] conjugated with AlexaFlour@546, all in concentration 1 µg/mL. The stained sample was incubated for 1 h at room temperature then washed and resuspended in FACS buffer for further analysis using BD FACS Calibur.

The size and the size distribution of both Ex and LUT-Ex were determined using Zetasizer (Malvern Instruments, UK) after their appropriate dilution with water. Additionally, their morphology was examined using transmission electron microscopy (TEM) (Jeol, JEM-100 CX electron microscope, Tokyo, Japan) after being stained with 1% w/v phosphotungstic acid. Their total protein content was quantified using the BCA protein assay kit (Sigma-Aldrich, USA).

##### 2.2.4.1. Determination of % entrapment efficiency (% EE) for LUT-Ex

For the determination of LUT % EE, the method reported by Aqil et al. (2017) was adopted with slight modifications. The prepared dispersion of LUT-Ex was subjected to centrifugation at 10,000 rpm for 10 min at 4 °C to remove the free unentrapped drug. The concentration of the precipitated drug was quantified spectrophotometrically at 350 nm and % EE was calculated using the following equation:

$$\text{\% Entrapment efficiency}=\frac{\text{initial amount of LUT added }-\text{free unentapped LUT}}{\text{initial amount of LUT }}\times 100$$

##### 2.2.4.2. *In vitro* drug release

The *in vitro* release of LUT from LUT-Ex versus LUT suspension was carried out using dialysis bag method in PBS (pH 7.4) containing 0.5% Tween® 80 (Elnaggar et al., 2018). Aliquots equivalent to 0.56 mg LUT were taken from either LUT-Ex or LUT suspension (prepared in methanol/PBS 1:9, respectively) and were inserted in dialysis bags (Visking® 36/32, 24 mm, MWCO 12,000–14,000, Serva, USA). After that, the bags were immersed in the release medium and kept in a shaking water bath (Kottermann, type 3047, Hanigsen, Germany) at 37 °C, 100 rpm. At different time intervals, samples were withdrawn from the release medium followed by substitution with an equal volume of fresh medium to maintain sink conditions. Finally, the concentration of LUT in the collected samples was determined spectrophotometrically at 350 nm. The study was performed in triplicate and results were expressed as mean ± SD.

##### 2.2.4.3. *In vitro* cellular uptake of coumarin-6 loaded exosomes (Cou-6-Ex)

A human hepatoma cell line (HEP-G2) (ATCC^®^ HB-8065™) was grown in DMEM-high glucose enriched with (10% v/v) FBS and antibiotics (100 U/mL penicillin 100 μg/mL streptomycin). Cells were incubated at 37 °C in a 5% CO_2_ incubator.

For evaluation of their cellular uptake, the isolated exosomes were fluorescently labeled using coumarin-6 as a lipophilic florescent dye (Liang et al., 2021; Kumar et al., 2022). In brief, equal volumes of exosomal dispersion in PBS and ethanolic dye solution (250 µg/mL) were incubated at 37 °C for 30 min. Cou-6-Ex or free ethanolic Cou-6 solution (final concentration 100 ng/mL) were incubated with Cells (2 × 10^4^ cells/well) in a 6-well plate (Corning Costar Corp., MA, USA) for 4 h. Afterwards, cells were rinsed with PBS, fixed with 4% paraformaldehyde, rinsed again with PBS, and stained with Hoechst 33342. Imaging was performed using confocal laser scanning microscopy (LEICA, DMi8, Mannheim/Wetzlar, Germany). Coumarin-6 was observed through the green channel at an excitation wavelength of 485 nm while Hoechst-nuclear counter stain was observed through the blue channel at 430 nm. Finally, the fluorescence intensity in the obtained images was quantified using Image J software (version 1.45 s) as previously described (Ashour et al., 2021; Shehata et al., 2022).

#### 2.2.5. *In vivo* studies

##### 2.2.5.1. Animals

The study was performed on male Sprague–Dawley rats (7–8 weeks, 180–220 g) housed in stainless steel mesh cages in 5 groups each of seven rats, under standard conditions of light and dark cycle illumination, relative humidity, and temperature, and they had free access to standard laboratory food and water throughout the study. Ethical approval for the study protocol was attained from the Medical Ethics Committee of Alexandria, Faculty of Medicine (IRB NO: 00012098-FWA NO: 00018699; membership in International Council of Laboratory Animal Science Organization, ICLAS).

##### 2.2.5.2. Induction of liver fibrosis

In order to induce liver fibrosis, rats were intraperitoneally injected with carbon tetrachloride (CCl_4_) diluted 1:1 with liquid paraffin in a dose of 1 mL/kg body weight. It was administered twice weekly for 2 months. The tested groups were; negative control (healthy rats IP injected with saline), positive control group (rats with induced liver fibrosis and left untreated), Ex-treated group (rats with liver fibrosis receiving a single dose of blank exosomal dispersion in PBS (450 µg/kg) via IP injection), LUT-treated group (rats with liver fibrosis receiving a single IP injection of LUT suspension in a dose of 1.4 mg/kg) and finally, the LUT-Ex treated group (rats with liver fibrosis receiving a single IP injection of LUT-Ex (the dose of LUT and Ex were 1.4 mg/kg and 450 µg/kg, respectively).

Rats were sacrificed 1-month post-treatment to collect blood samples from the retro-orbital venous plexus. The collected blood samples were then centrifuged at 4,000 rpm for 10 min to isolate serum samples for biochemical assessment of various hepatic function markers. Additionally, liver tissues were taken from dissected animals, rinsed with ice-cold saline, and weighed to determine % relative liver weight to body weight. Then, a small portion from each liver sample was fixed in formalin (10% v/v) for histopathological examination and another portion was snap-frozen and used for the biochemical determination of different profibrotic and inflammatory markers.

##### 2.2.5.3. Assessment of hepatic function

The levels of the different liver function markers were colorimetrically determined in the isolated serum samples as alanine transaminase (ALT), aspartate aminotransferase (AST) and alkaline phosphatase (ALP) according to the manufacturer’s instructions of the suitable assay kits (Bessey et al., 1946; Reitman & Frankel 1957).

##### 2.2.5.4. Assessment of inflammatory and profibrotic markers

The levels of the inflammatory cytokine; tumor necrosis factor-alpha (TNF-α) as well as different hepatic profibrotic markers (hydroxyproline (HP), transforming growth factor beta (TGF-β) and matrix metalloproteinase-2 (MMP2) were determined in the liver tissue homogenate using ELISA method following the manufacturer’s instructions.

##### 2.2.5.5. Histopathological examination

Isolated liver specimens were fixed with 10% v/v formalin and then further processed to obtain 5 µm thick sections. The obtained sections were stained with hematoxylin, eosin (H&E) and Masson’s trichrome stains. Sections were then examined under a light microscope equipped with a digital camera (Olympus America Inc., USA). Additionally, for Masson’s trichrome-stained sections, the amount of collagen fibers (green stain) was quantitatively analyzed using ImageJ software (version 1.45 s)

### 2.3. Statistical analysis

All Experiments were carried out in triplicate and data were expressed as means ± SD. Statistical analysis of the results was performed using either unpaired *t* test or one-way analysis of variance (ANOVA) followed by Tukey’s test for multiple comparisons (GraphPad Prizm software, version 7.0). The significance level was set at *p* ≤ 0.05.

## 3. Results and discussion

### 3.1. Isolation and characterization of bone marrow-MSCs

**Figure 1.**
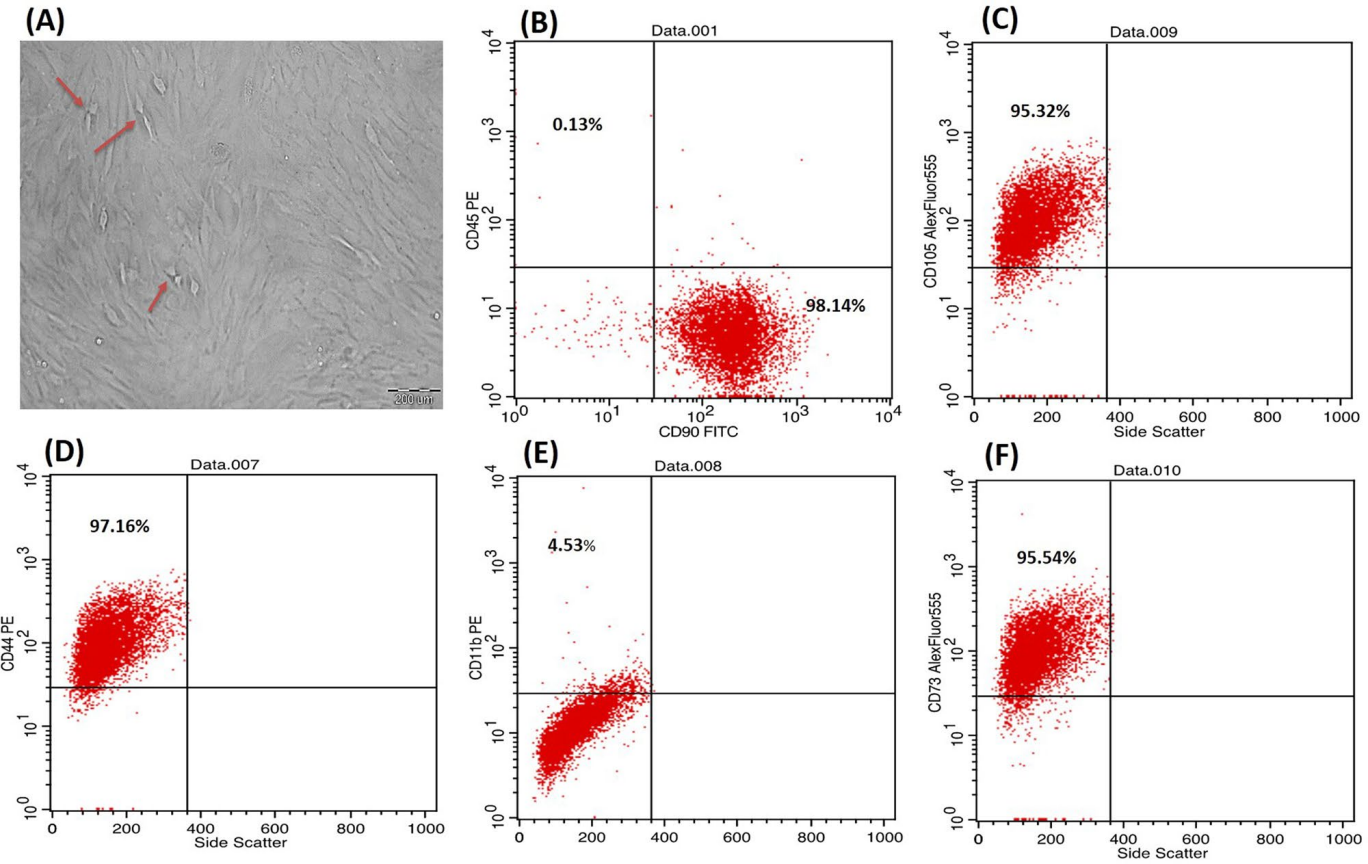
Phase contrast inverted light microscope, morphological characteristics of bone marrow-MSCs showing passage 3, 90% confluent spindle cells, red arrows pointing on proliferating cells (**A**) at magnification ×200; scale bar represents 200 nm. Flow cytometric analysis of cell-surface markers of bone marrow-MSCs at passage 3 (**B–F**) showing that 98.14% of the cultured cells expressed the mesenchymal cell marker CD90 cells and were almost negative for the CD45 hematopoietic marker (0.13%) (**B**), 95.32% for CD105 (**C**), 97.16% for CD44 (**D**), 4.5% for hematopoeitic marker CD 11b (**E**) and 95.54% for CD 73 (**F**). Upper left quadrant represents the scatter for the cells expressing the CD marker blotted on *Y* axis, while lower right quadrant represents the scatter for the cells expressing the CD marker blotted on *X* axis.

P3 bone marrow-MSCs showed a typical spindle shape adherent proliferating cells, forming colonies while growing and a monolayer when confluent (Figure 1A). Whereas Figure 1B–F showed the immunophenotyping results which revealed the expression of mesenchymal surface markers CD44, CD90, CD105 and CD73 with 97.16%, 98.14%, 95.54% and 95.32%, respectively. While they were almost negative to the hematopoietic surface markers CD45 and CD11b showing only 0.13% and 4.53%, respectively.

### 3.2. Isolation of exosomes and their in-vitro characterization

**Figure 2.**
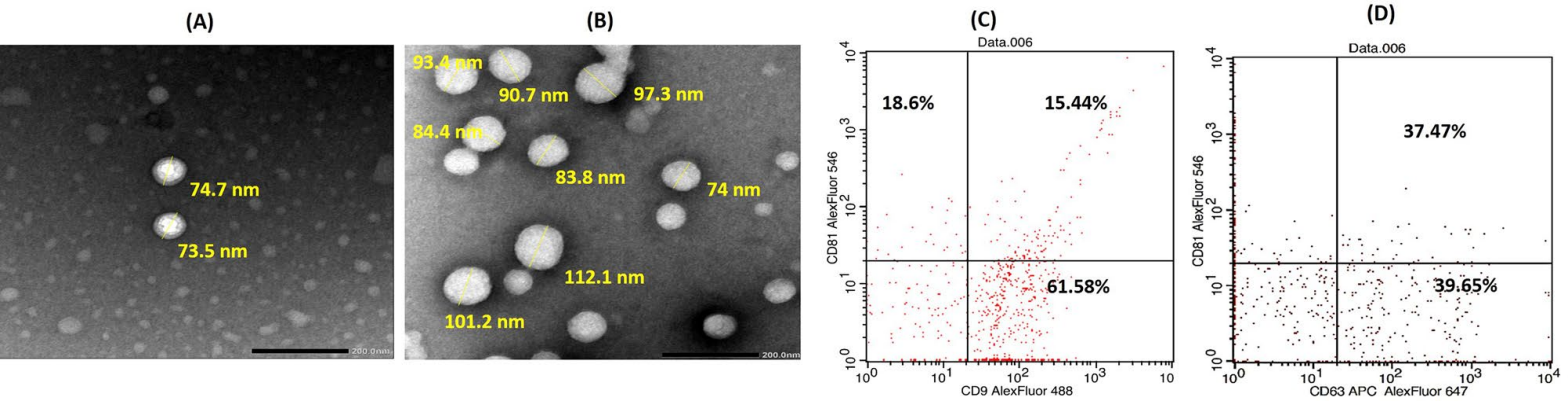
TEM images of blank exosomes **(A)** and Luteolin loaded exosomes **(B)** at magnification ×40 K; scale bar represents 200 nm. Flow cytometric analysis of exosomes surface tetraspanins markers **(C, D)**; where upper left quadrant of scatter blots represents exosomes expressing the CD marker blotted on *Y* axis, while lower right quadrant represents exosomes expressing the CD marker blotted on *X* axis and the upper right quadrant represents the exosomes expressing both CD markers.

Exosomes were isolated from bone marrow MSCs as previously described. Particle size analysis revealed that they have a small size (89.4 ± 7.5 nm) with a narrow size distribution (PDI = 0.31 ± 0.001). Additionally, they have a negative ζ-potential value of −30 ± 1.13 mV, confirming their colloidal stability. Their negative ζ-potential value was mainly ascribed to their negatively charged phospholipid membrane (Kim et al., 2016; Donoso-Quezada et al., 2020). The obtained colloidal properties of the isolated exosomes were congruent with those previously reported (Donoso-Quezada et al., 2020). The morphological analysis by TEM illustrated typical vesicular-shaped particles (Figure 2A) of sizes that almost matched those obtained by the dynamic light scattering technique. Furthermore, the total protein content of the exosomes, determined using BCA assay, was 900 µg/mL, reflecting their rich content as well as the efficiency of the adopted isolation procedure (Thabet et al., 2020). The expression of tetraspanins on exosomes demonstrated that the percentages of CD9, CD63 and CD81 were 77.02%, 77.12% and 34.04%, respectively (Figure 2C,D).

### 3.3. Preparation and optimization of LUT-Ex

In the current work, LUT (a phytomedicine with a well-known antifibrotic activity (Domitrović et al., 2009; Li et al., 2015) was loaded into bone marrow MSCs-derived exosomes for the first time aiming to increase its therapeutic potential in the management of liver fibrosis.

For optimization of drug loading, LUT was incorporated into exosomes using two different methods either physical incubation or mild sonication. It was found that luteolin % EE was 3%±0.1 and 40%±1.32 for physical incubation and sonication methods, respectively. This corroborated with what was previously reported for the use of exosomes as a drug delivery carrier where sonication facilitates drug diffusion into exosomes via reorganization of their membrane structure leading to high drug loading (Kim et al., 2016; Li et al., 2020). Accordingly, the sonication method was adopted in the present study to achieve an efficient drug loading capacity.

The colloidal properties of the prepared LUT-Ex were as follows: size; 150 ± 4.3 nm, PDI; 0.3 ± 0.01, ζ-potential; −28.1 ± 2.1 mV. The observed increase in size upon sonication came in line with previous studies and could be assigned to possible reformation of exosomes after sonication (Kim et al., 2016; Salarpour et al., 2019). Additionally, it was found that there was no change in either ζ-potential or morphology (Figure 2B) of exosomes after LUT loading, confirming efficient entrapment of the drug in exosomes and not being just attached to their surfaces. The protein content was maintained after drug loading, indicating that the integrity and composition of exosomes were not affected by LUT loading. This could be attributed to the ability of LUT, as a plant flavonoid, to stabilize the lipid membranes (Samanta et al., 2011). Similar observations were reported for other flavonoids such as quercetin (Qi et al., 2020) and curcumin (Kalani et al., 2016) where the properties of exosomes were not influenced by drug loading.

### 3.4. *In vitro* drug release

**Figure 3.**
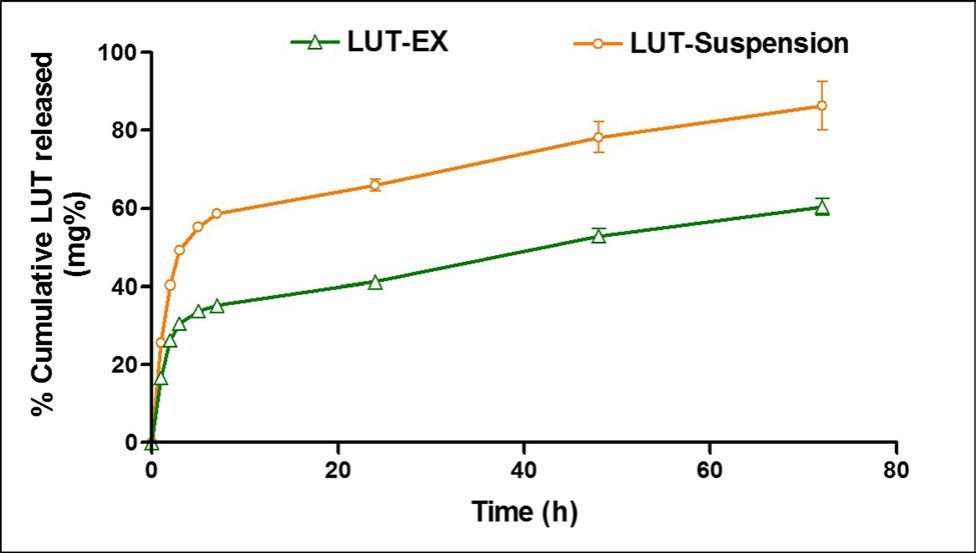
Release profiles of LUT-Ex and LUT suspension at 37 °C in PBS + 0.5% w/v Tween^®^ 80. Data presented as means ± SD (*n* = 3).

Figure 3 demonstrated the in-vitro drug release profile of LUT-Ex versus LUT suspension. LUT suspension demonstrated a relatively rapid drug release profile where approximately 50% LUT was released after 3 h and 86% released after 72 h. In contrast, LUT-Exo exhibited a significantly lower % cumulative release with only 60% released after 72 h suggesting the ability of the exosomes to allow for a sustained LUT release (*p* ≤ 0.05). Additionally, loading of LUT into exosomes resulted in a significant reduction in initial drug release (∼30% after 3 h) (*p* ≤ 0.05). The rapid release of LUT from loaded exosomes in the first 3 h may be attributed to the membrane-bound portion of the drug (Massey et al., 2021). Similar controlled release profiles were previously reported in literature for various drugs incorporated into exosomes that was ascribed to the efficient internalization of the drug inside the core of exosomes (Kim et al., 2016; Li et al., 2020).

### 3.5. *In vitro* cellular uptake of Cou-6-Ex

**Figure 4.**
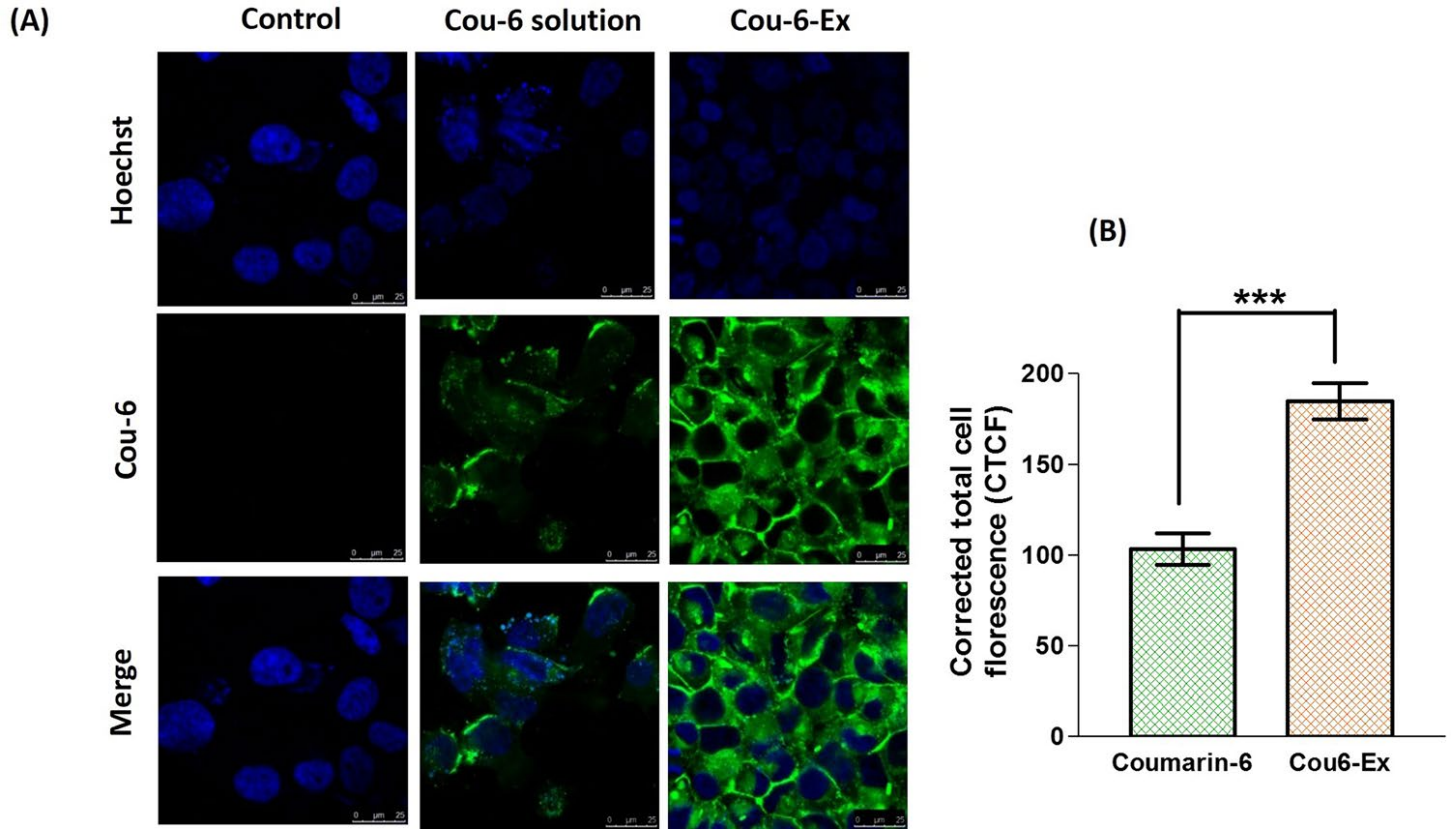
Cellular uptake of coumarin-6-exosomes versus free coumarin-6 solution in HEP-G2 cells. Green and blue fluorescence signals signify coumarin-6 and the Hoechst-stained cellular nuclei, respectively. (A) Images obtained from the confocal laser scanning microscope after 4 h (magnification 63×). (B) Calculated corrected total cell fluorescence (CTCF) obtained from ImageJ analysis presented as mean ± SEM. Data were analyzed using unpaired *t* test. *** denotes the highly statistically difference between coumarin-6-exosomes and coumarin-6 solution at *p* ≤ 0.001.

The uptake ability and effective internalization of compounds by cells is considered as a vital parameter when developing novel nanosystems (Donoso-Quezada et al., 2020). The *in vitro* cellular uptake of exosomes was reported to start after 30–40 min from their incubation, reaching the maximum accumulation after ∼3 h (Donoso-Quezada et al., 2020). Accordingly, Cou-6-labeled exosomes were used to evaluate their *in vitro* uptake in HEP-G2 cell line after 4 h incubation. The results show a significant (*p* ≤ 0.001) increase in the fluorescence intensity for Cou-6-Ex by 78.4% compared to free Cou-6 solution suggesting a higher cellular uptake (Figure 4A,B). Comparable results have been previously obtained for enhanced uptake of Bodipy TR Ceramide-labeled exosomes in different cell lines (Donoso-Quezada et al., 2020). The higher cellular uptake of exosomes could be mainly due to their efficient cellular internalization via both clathrin-mediated endocytosis and micropinocytosis (Donoso-Quezada et al., 2020).

### 3.6. *In vivo* evaluation of hepatic anti-fibrotic activity

In the present study, liver fibrosis was induced in rats using CCl_4_ which initiates many cytotoxic effects leading eventually to sever liver damage (Ashour et al., 2021).

The calculated % relative liver weight to body weight at the end of the study demonstrated the severe hepatic impairment in the positive control group (the highest value) as compared to other groups (Table 1). Additionally, it was found that all the treatments (LUT suspension, Ex and LUT-Ex) significantly decreased the % relative liver weight compared to positive control group (*p* ≤ 0.05) confirming their antifibrotic activity. However, the different treatments did not return the % relative liver weight back to normal as indicated by the significant difference with the negative control group (*p* ≤ 0.05) (Table 1).

**Table 1.** Effect of blank or LUT-loaded exosomes versus LUT suspension on % relative liver weight to body weight and markers of liver function after IP single dose administration (1.4 mg/kg) to CCl_4_-induced fibrotic rat model.

Treatment group	% Relative liver weight	ALT (U/mL)	AST (U/mL)	ALP (U/mL)
Negative control	2.9^c^±0.5	54.2^e^±2.9	80.2^c^±5.5	208.7^d^±12.4
Positive control	4.4^a^±0.5	95.8^a^±5.1	112.22^a^±6.3	368.9^a^±10.3
LUT suspension	3.7^b^±0.1	65.5^c^±3.1	92.4^b^±3.6	255.6^c^±9.6
Ex	3.6^b^±0.4	73.7^b^±4.4	105.8^a^±4.7	321.4^b^±11.4
LUT-Ex	3.4^b^±0.2	59.1^d^±2.8	84.2^c^±6.5	216.5^d^±8.2

The study was conducted on male Sprague–Dawley rats of 7 animals in each group (*n* = 7). Values were expressed as mean ± SD. Data were analyzed using one-way ANOVA followed by post hoc test (Tukey’s test) for group comparisons. Means of similar symbols are statistically insignificant, *a* >*b*>*c*>*d* (*p* ≤ 0.05).

#### 3.6.1. Assessment of hepatic function

Table 1 shows the measured serum markers for liver function evaluation of the different study groups. The levels of all tested markers were significantly elevated in the positive control group compared to the negative control (*p* ≤ 0.05), indicating remarkable hepatocellular damage (El-Mezayen et al., 2018). On the other hand, the single IP administration of different treatments succeeded to alleviate the induced hepatic fibrosis in the following order: EX < LUT suspension < LUT-Ex as evidenced by the reduction in the levels of the evaluated markers. The improvement in hepatic function for the exosomes group was attributed to the ability of MSCs-derived exosomes to ameliorate liver fibrosis via their cargo that is derived from the parent cell. This cargo would carry the same anti-inflammatory, immunomodulatory and regenerative properties of MSCs, thus was able to reduce hepatic inflammation and collagen deposition and therefore protects hepatocytes (Li et al., 2013). Similarly, Jiang et al. (2018) ascribed the hepatoprotective activities of human umbilical cord MSCs-derived exosomes in a mouse model for acute and chronic liver injury to their antioxidant and anti-apoptotic activity. Furthermore, Shao et al. (2020) described the ability of exosomes secreted by human umbilical cord MSCs to inhibit macrophage activation hence could treat the induced liver injury via suppressing IL-6 signaling pathway. With respect to LUT-treated rats, LUT caused a significantly higher reduction in the levels of ALT, AST and ALP compared to both positive control and blank exosomes groups (*p* ≤ 0.05) (Table 1). This was assigned to the well-documented antifibrotic activity of LUT mediated via enhancing the degradation of extracellular matrix in fibrotic liver along with improving the capability for hepatic regeneration (Domitrović et al., 2009). Additionally, Li et al. (2015) further demonstrated that LUT reversed liver fibrosis through suppression of proliferation, migration, collagen synthesis and expression of genes related to fibrosis in the activated HSC. Moreover, LUT was proven to stimulate apoptosis to activated HSC (Li et al., 2015). The highest activity was for the LUT-Ex group which verified the ability of the exosomes to augment the antifibrotic activity of LUT. Exosomes can effectively fuse with cells hence promoting efficient internalization of drugs in the target cells to exert their activities (Wang et al., 2021). It is worth mentioning that only the administration of a single dose of LUT-Ex (1.4 mg/kg) returned the values of tested AST and ALP back to normal as confirmed by the statistical similarity to the negative control group (*p* > 0.05). Accordingly, the elaborated LUT-Ex in this work might be regarded as a good choice to enhance the therapeutic potential of LUT at the low administered dose.

#### 3.6.2. Assessment of hepatic inflammatory markers

Elevation in the levels of TNF-α and its receptors is a characteristic hallmark in liver fibrosis (Tacke et al., 2009). Following liver injury, this inflammatory cytokine is produced by Kupffer cells and accelerates the activation of HSC (Tacke et al., 2009). Therefore, the positive control group exhibited a significant increase in TNF-α protein levels when compared to the negative control group (*p* ≤ 0.001) (Table 2). In contrast, other study groups (LUT suspension, blank exosomes and LUT-Ex) revealed a significant decrease in the protein levels of the measured inflammatory marker when compared to the positive control group (*p* ≤ 0.001). LUT suspension group showed 35.5% decrease in the level of TNF-α compared to the positive control group (*p* ≤ 0.001) (Table 2). This might elucidate the underlying antifibrotic mechanism of LUT through suppression of inflammation (Balamurugan & Karthikeyan, 2012; Tai et al., 2015; Park & Song, 2019). With respect to blank exosomes group, the level of TNF-α was significantly decreased by 27.8% compared to positive control group (*p* ≤ 0.001) (Table 2). This was attributed to the reported immunomodulatory function of exosomes isolated from MSCs through inhibiting the secretion of TNF-α by macrophages (Al-Khawaga & Abdelalim, 2020). Interestingly, the administration of LUT-Ex brought about the highest reduction in the value of the tested marker (46% decrease) when compared to the positive control group (*p* ≤ 0.001) (Table 2). The previous finding strongly confirmed the important role of exosomes in augmenting the anti-inflammatory potential of the incorporated LUT.

**Table 2.** Effect of blank and LUT-loaded exosomes versus LUT suspension on both hepatic inflammatory and profibrotic markers after single dose IP administration (1.4 mg/kg) to CCl_4_-induced fibrotic rat model.

Treatment groups	Inflammatory marker	Profibrotic markers		
	TNF-α (pg/mg protein)	HP (µg/gm tissue)	TGF-β (pg/ml)	MMP2 (ng/ml)
Negative control	33.3^e^±4.6	112.8^e^±5.7	198.7^e^±11.6	5.3^e^±0.01
Positive control	78.3^a^±3.5	243.5^a^±8.7	410.3^a^±8.0	8.1^a^±0.1
LUT suspension	50.5^c^±1.3	139.0^c^±3.4	290.3^c^±2.5	6.7^c^±0.01
Ex	56.5^b^±3.1	155.3^b^±8.1	315.7^b^±7.8	7.1^b^±0.1
LUT-Ex	42.3^d^±3.1	128.8^d^±5.1	262.3^d^±7.5	6.0^d^±0.1

The study was conducted on male Sprague–Dawley rats of 7 animals in each group (*n* = 7). Values were expressed as mean ± SD. Data were analyzed using one-way ANOVA followed by post hoc test (Tukey’s test) for group comparisons. Means of similar symbols are statistically insignificant, *a > b > c > d > e* (*p* ≤ 0.001).

#### 3.6.3. Evaluation of hepatic profibrotic markers

It is well known that liver fibrosis is accompanied by the increase in the levels of many profibrotic markers such as HP, MMP-2 and TGF-β (Ashour et al., 2021). HP is an important constituent for the collagen matrix produced by activated HSC (Ashour et al., 2021). Additionally, MMP-2 (a gelatinase enzyme) (Duarte et al., 2015) and TGF-β (Gressner et al., 2002) were implicated in fibrosis progression by increasing the proliferation of HSC at the initial stages of the disease. Consequently, positive control group exhibited a significant increase in levels of both HP and TGF-β by 1.1-fold, whereas MMP-2 increased by 52.8% when compared to negative control group (*p* ≤ 0.001) (Table 2). Conversely, LUT suspension, blank exosomes and LUT-Ex significantly decreased the values of the tested markers to variable degrees compared with positive control group (blank exosomes < LUT suspension < LUT-Ex, *p* ≤ 0.001). The observed reduction in the values of the measured markers for LUT suspension group (42.1%, 29.3% and 17.2% decrease in HP, TGF-β and MMP-2, respectively versus positive control (Table 2) was mainly ascribed to its reported antifibrotic potential (Domitrović et al., 2009; Li et al., 2015). Regarding blank exosomes, the levels of the tested markers reduced by 36.2%, 23.1% and 12.3% for HP, TGF-β and MMP-2, respectively versus positive control group. The observed alleviation of the induced fibrosis was due to the previously described antifibrotic activity of MSCs-derived exosomes (Li et al., 2013; Jiang et al., 2018; Shao et al., 2020). LUT-Ex revealed a surpassing antifibrotic activity as indicated by the greatest reduction in HP, TGF-β and MMP-2 by 47.1%, 36.1% and 25.9%, respectively versus positive control group (Table 2). The remarkable efficacy observed for LUT-Ex highlighted the effect of exosomes on potentiating the activity of the loaded LUT.

#### 3.6.4. Histopathological assessment of liver fibrosis

**Figure 5.**
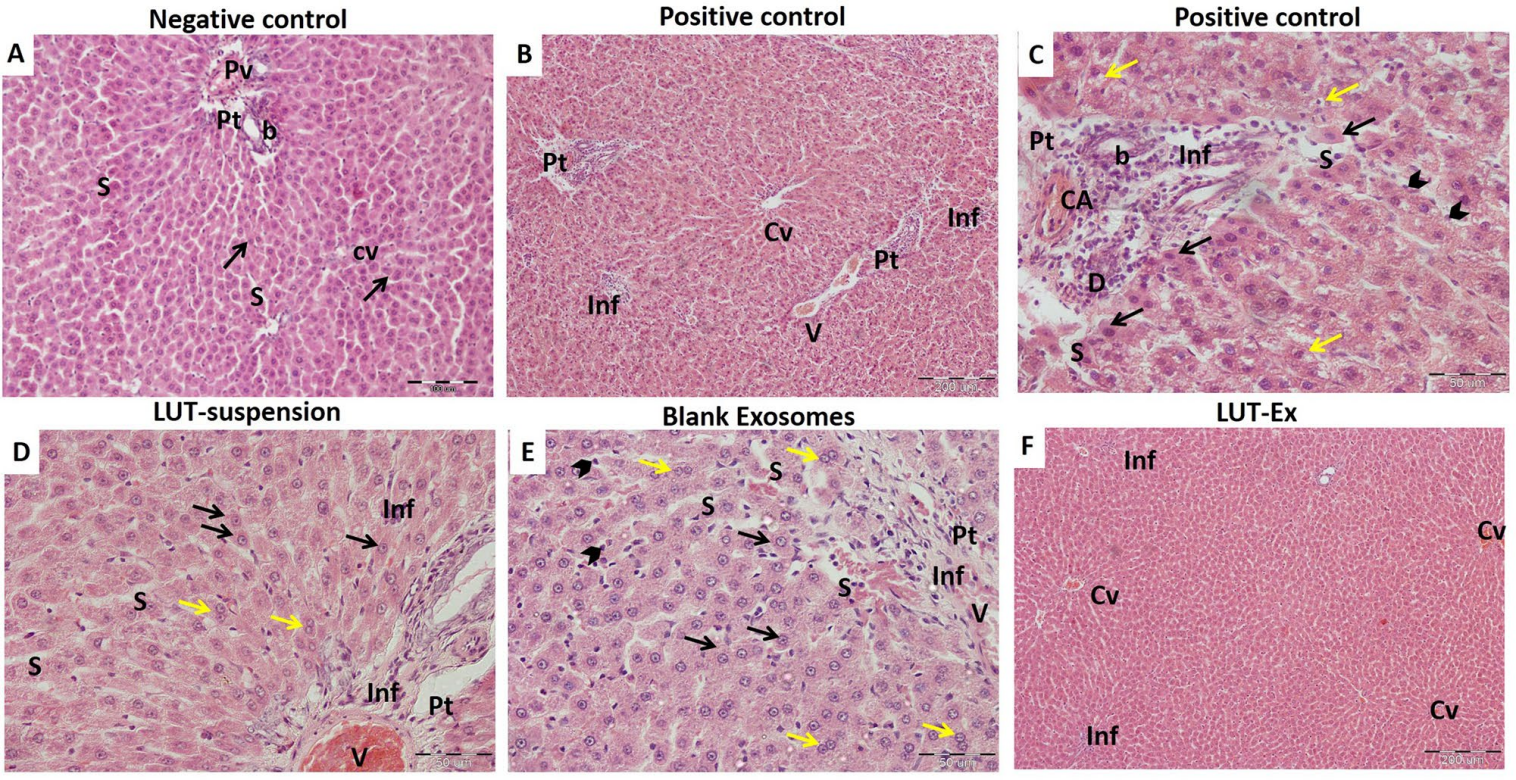
Hematoxylin and eosin (H&E) staining of liver tissue of the different studied groups. (**A**) Negative control group showed classical hepatic architecture with branching anastomosing cords of hepatocytes one or two cell thickness radiating from central veins (Cv) and separated from each other by narrow sinusoids (S). The hepatocytes showed eosinophilic granular cytoplasm with vesicular nuclei and prominent nucleoli (arrow). The Portal tracts (Pt) with branches of portal veins (Pv) and bile ducts (b) were also seen. (B,C) Positive control group revealed extensive liver damage. (B) A low-power image showing several areas of mononuclear cellular infiltration, either in-between the hepatocytes (Inf), surrounding the central vein (Cv), or within the portal tracts (Pt), leading to disruption of the hepatic architecture. Congested portal vein is also noticed (V). (C) A high-power image demonstrating a marked hepatocellular degeneration. Many hepatocytes are seen with hyper-eosinophilic cytoplasm, loss of cytoplasmic granularity and dark nuclei (black arrows). Others are seen with rarified cytoplasm and dense irregular nuclei (yellow arrows). The portal tracts (Pt) are expanded by mononuclear cellular infiltration (Inf), with proliferation and dilatation of the bile ducts (b) and congested hepatic arterioles (CA). The hepatic sinusoids (S) are dilated and lined by many Kupffer cells (arrow heads). (D) LUT-suspension group illustrated showed restoration of the normal appearance of the hepatocytes with their pale large nuclei (black arrows). Moreover, many of them are binucleated (yellow arrows). Areas of mononuclear cellular infiltration (Inf) are still apparent in-between the cells or within the portal tract (Pt). The portal vein (V) and the hepatic sinusoids (S) are dilated and congested. (E) Plain exosomes group presented mild hepatic improvement. Hepatocytes appear more or less normal with slightly eosinophilic granular cytoplasm and large central vesicular nuclei with prominent nucleoli (black arrows). Additionally, numerous binucleated hepatocytes can be detected (yellow arrows). Areas of mononuclear cellular infiltration (Inf) are still seen within the portal tract (Pt) around the congested branch of the portal vein (V). The hepatic sinusoids (S) are dilated and congested. Many Kupffer cells are also noticed (arrow heads). (F) LUT-Ex group exhibited a prominent restoration of the liver architecture. The hepatocytes are apparently normal and arranged in cords 1–2 cell thickness. Limited sporadic regions of mononuclear cellular infiltration (Inf) are seen in-between the hepatocytes. Some central veins (Cv) are congested (H&E stain, Mic. Mag. A: ×200, B, F: ×100, C, D, E: ×400).

Histopathological examination of liver tissue for all study groups was conducted to further confirm the obtained results. Isolated liver sections were stained with H & E stain for the evaluation of any histopathological changes. The negative control group (Figure 5A) showed well-organized hepatic architecture with branching anastomosing cords of hepatocytes radiating from central veins and separated from each other by narrow sinusoidal spaces. Whereas the positive control group revealed extensive damage with disturbance of hepatic architecture (Figure 5B,C). Several areas of mononuclear cellular infiltration, either in-between the hepatocytes, surrounding the central vein or within the portal tracts were detected (Figure 5B). Additionally, marked hepatocellular degeneration was verified by the presence of many hepatocytes with hyper-eosinophilic cytoplasm, loss of cytoplasmic granularity and dark nuclei. Others were seen with rarefied cytoplasm and dense irregular nuclei. Dilated sinusoids lined with many Kupffer cells were also noticed (Figure 5C). The LUT-suspension group showed mild hepatic degeneration with the restoration of the normal appearance of the hepatocytes confirming the hepatoprotective effect of the drug (Figure 5D). Blank exosomes group exhibited some correction for the induced fibrosis with the appearance of some areas of mononuclear cellular infiltration especially within the portal tract and around the congested branch of the portal vein. Additionally, some hepatocytes were observed to be more or less normal (Figure 5E). Interestingly, LUT-Ex group showed a prominent restoration of the liver architecture with rarely noticed signs of degeneration (Figure 5F). This finding further ensured the superiority of the prepared LUT-Ex in resolution of the induced fibrosis and matched the obtained results of biochemical tests.

**Figure 6.**
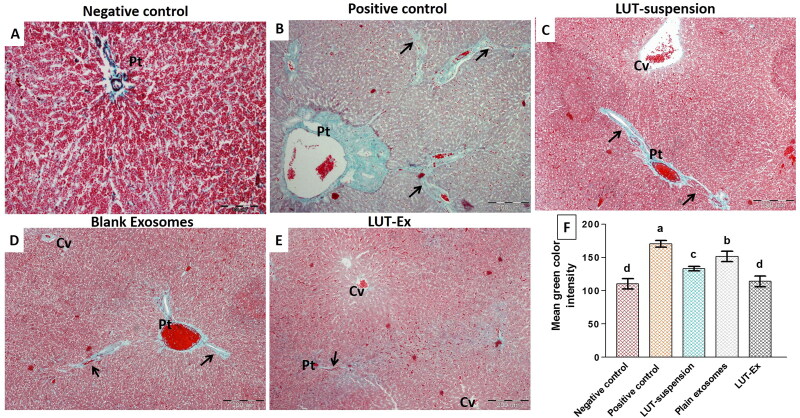
(A–E) Masson’s trichrome staining of the different tested groups. (F) Graphical representation of the quantified amount of collagen fibers using image J software. (A) Negative control group revealed minimal collagen fibers mainly around portal tracts (Pt). (B) Positive control group showed excessive collagen fibers deposition and formation of thick connective tissue septa (arrows) that are radiating from the portal tract (Pt), resulting in evident pseudo-lobulation of the liver. (C,D) LUT suspension and plain exosomes groups, respectively, demonstrated moderate improvement with formation of the fibrous tissue and septation (arrows) especially surrounding the portal tracts (Pt) and central veins (Cv). (E) LUT-Ex group presented the greatest antifibrotic activity with mild collagen deposition around the portal tracts (Pt) and central veins (Cv). Septa are few and thin (arrow) (Masson’s trichrome stain, Mic. Mag. A: ×200, B, C, D, E: ×100). (F) Graphical representation showing quantification of amount of collagen deposition (indicated by mean green color intensity) for different test groups. Values were presented as means ± SD (*n* = 7). Data were statistically analyzed using one-way ANOVA followed by post hoc test (Tukey) for multiple comparisons. Means of similar symbols were statistically insignificant: *a* > *b* > *c* > *d* (*p* ≤ .05).

To further demonstrate the deposition of collagen fibers, a key parameter for liver fibrosis, liver specimens from different study groups were also stained with Masson’s trichrome to stain collagen fibers with green color (Figure 6A–E). The negative control group revealed minimal collagen fibers mainly around portal tracts (Figure 6A). The positive control group illustrated excessive collagen fibers deposition and formation of thick connective tissue septa radiating from the portal tract resulting in evident pseudo-lobulation (Figure 6B). Both LUT suspension and blank exosomes groups showed moderate improvement with moderate formation of the fibrous tissue and septation especially surrounding the portal tracts and central veins (Figure 6C,D). On the other hand, the administration of LUT-Ex succeeded to achieve a remarkable antifibrotic activity characterized by slight collagen deposition around the portal tracts and central veins and the presence of few thin septa (Figure 6E).

For more clarification, the intensity of the observed green color (an indicator for the amount of deposited collagen) was quantified using image J software (version 1.45 s) (Figure 6F). The positive control group demonstrated the maximum deposition of collagen fibers evidenced by the significantly highest mean green color intensity compared to all treatment groups (*p* ≤ 0.01). Both LUT-suspension and blank exosomes groups revealed a significant decrease (*p* ≤ 0.01) in mean green color intensity compared to the positive control group, proving their efficacy in reducing fibrous tissue formation. However, the activity of LUT-suspension was higher than that of blank exosomes as indicated by the significant difference between them in the measured color intensity (*p* ≤ 0.05). On contrary, LUT-EX group demonstrated the least deposition of collagen fibers as indicated by the significantly least mean color intensity compared with other groups (*p* ≤ 0.01). Interestingly, LUT-Ex succeeded to restore the hepatic state back to normal as indicated by the insignificant difference with the negative control group (*p* > 0.05).

In conclusion, the obtained results of both biochemical and histopathological evaluation proved the superior antifibrotic potential of LUT-Ex versus LUT-suspension which could be attributed to the biological activity of blank exosomes hence augmenting drug activity. Additionally, the ability of exosomes to facilitate LUT penetration through liver cells paving the way to exert its effect for a longer time.

## 4. Conclusion

Herein, for the first time, LUT-Ex was prepared and its efficacy in the treatment of CCL_4_-induced liver fibrosis was assessed versus either LUT-suspension or blank exosomes. A simple preparation method was employed based on sonication. The elaborated LUT-Ex offered many advantages as allowing for deep drug tissue penetration due to their small size, sustained drug release, long circulation time, safety and biocompatibility. Additionally, the effect of bone marrow-MSC-derived exosomes in augmenting drug effect via their inherent antifibrotic, antioxidant, immunomodulatory and regenerative activity was verified by the observed outstanding therapeutic potential of the developed LUT-Ex. Accordingly, LUT-Ex could be considered as a promising bio-inspired biocompatible nanosystem for liver fibrosis therapy.
